# LHRH-Conjugated Magnetite Nanoparticles and Nanorods as Magnetic Resonance Imaging Contrast Agents for Targeting Triple-Negative Breast Cancer

**DOI:** 10.3390/jfb17030134

**Published:** 2026-03-10

**Authors:** Chukwudi C. Ezeala, John D. Obayemi, Ali A. Salifu, Theresa Ezenwafor, Yiporo Danyuo, Maria Chinyerem Onyekanne, Stanley C. Eluu, Toyin Aina, Josephine Oparah, Precious O. Etinosa, Olushola S. Odusanya, Winston O. Soboyejo

**Affiliations:** 1 Department of Materials Science and Engineering, African University of Science and Technology, Km 10 Airport Road, Galadimawa, Abuja 900107, Nigeria; cezeala@aust.edu.ng (C.C.E.);; 2 Department of Mechanical and Materials Engineering, Worcester Polytechnic Institute, 100 Institute Road, Worcester, MA 01609, USA; 3 Department of Biomedical Engineering, Worcester Polytechnic Institute, Gateway Park Life Sciences and Bioengineering Centre, Worcester, MA 01605, USA; 4 Department of Engineering, Boston College, 245 Beacon Street, Chestnut Hill, MA 02467, USA; 5 Department of Mechanical Engineering, Academic City University, Property #79-30, Agbogba, Accra GE-189-5224, Ghana; 6 Department of Biotechnology, Ebonyi State University, Enugu-Abakaliki Road, Abakaliki 480214, Nigeria; 7 Department of Biomedical Engineering, College of Engineering, Afe Babalola University, Ado Ekiti, Km 8.5 Afe Babalola Way, Ado Ekiti 360102, Nigeria; 8 Department of Biotechnology, Sheda Science and Technology Complex, Abuja 904105, Nigeria; shola2@hotmail.com; 9 Department of Mechanical Engineering, State University of New York (SUNY Poly) Polytechnic Institute, 100 Seymour Street, Utica, NY 13502, USA

**Keywords:** magnetite nanoparticles (MNP), triple-negative breast cancer (TNBC), luteinizing hormone-releasing hormones (LHRH), functionalized magnetite nanoparticles, magnetic resonance imaging (MRI)

## Abstract

This paper presents the results of an experimental study on the effects of magnetite nanoparticle (MNP) shape on magnetic resonance imaging (MRI) of triple-negative breast cancer (TNBC) xenograft tissues. T_2_-weighted MRI scans using spherical shaped MNPs as contrast agents are compared to MRI scans done with nanorod-shaped MNPs as contrast agents. The MNPs were first coated with polyethylene glycol (PEG) and subsequently conjugated to luteinizing hormone-releasing hormone (LHRH) to specifically target LHRH receptors, which are present at high levels on the surfaces of xenograft TNBC cells/tissues. After 3 weeks of tumor growth in nude immunocompromised mice, LHRH-conjugated (functionalized) and unconjugated (non-functionalized) MNPs were injected into the immunocompromised mice. Four types of MNPs were used: non-functionalized nanorod-shaped MNPs (BMNR); LHRH-conjugated nanorod-shaped MNPs (LCMNR); non-functionalized spherical-shaped MNPs (BSSMNP); and LHRH-conjugated spherical-shaped MNPs (LCSSMNP). T_2_-weighted magnetic resonance imaging (MRI) scans were obtained from the mice before the injection of the MNPs and two hours after the injection of the MNPs. The results show that using nanorod-shaped LHRH-conjugate MNPs as contrast agents yielded higher-resolution T_2_-weighted MRI scans of TNBC tumors compared to those from spherical-shaped MNPs. The implications of the results are discussed in relation to potential applications of functionalized MNPs in MRI for TNBC diagnosis.

## 1. Introduction

Triple-negative breast cancer (TNBC) is an aggressive type of breast cancer that has a poor prognosis and poor clinical outcomes compared to other types of breast cancer [1,2]. TNBC does not express the common biomarkers estrogen receptor (ER), progesterone receptor (PR), and human epidermal growth factor receptor-2 (HER2), making it difficult for conventional treatment that works for other types of breast cancer [1,3,4]. TNBC accounts for 10–15% of the total breast cancer cases, and patients diagnosed with metastatic TNBC were shown to have a median survival of approximately 13 months [3]. TNBC has a high relapse rate within five years after treatment compared to other breast cancer types [5]. TNBC must be accurately diagnosed and subtyped at an early stage to enable timely intervention and appropriate treatment [4]. Magnetic resonance imaging is a highly sensitive method for breast cancer detection. It is often chosen for high-risk patients (patients with a family history of BRCA gene mutation) for its ability to detect breast cancer at an early stage compared to other methods of breast cancer detection, like mammography and ultrasonography [3].

Magnetic resonance imaging (MRI) is a non-invasive imaging technique that creates three-dimensional anatomical images used to detect diseases such as cancer and cardiovascular conditions [6,7]. MRI uses protons, which are abundant in biological tissues, to generate magnetic resonance (MR) images. These protons have intrinsic magnetic moments and can generate magnetic fields. Typically, the spins of individual protons in biological tissues are randomly oriented, resulting in a net magnetic moment of zero. MRI applies a strong, uniform external magnetic field to align the magnetic moments of protons to produce an equilibrium magnetization along the external magnetic field’s longitudinal axis [6,8]. This equilibrium magnetization is subsequently disrupted by introducing an external radio frequency (RF) pulse, which transfers energy to the protons by rotating their magnetic moments away from the longitudinal axis by 90° into the transverse plane, perpendicular to the external magnetic field, creating a net transverse magnetization [7,8]. When the RF pulse is switched off, the protons return to their resting alignment through various relaxation processes while emitting RF energy and revert to their default lower energy state; the net longitudinal magnetization regrows and returns to its original value (T_1_ relaxation); and the net transverse magnetization decreases or decays (T_2_ relaxation) as the protons dephase randomly in the transverse plane [8]. MRI generates images with varying contrast due to differences in relaxation times, T_1_ and T_2_.

Contrast agents are often used for MRI of breast cancer to enhance the contrast between normal and diseased tissues and to produce images that ensure easy detection, diagnosis, and staging of breast cancer. Contrast agents can be categorized as T_1_ or T_2_ agents based on their ability to shorten T_1_ or T_2_ relaxation times, respectively. MNPs have been developed as T_2_-weighted contrast agents owing to their favorable physicochemical properties [9,10,11]. MNPs are biodegradable [9], biocompatible [9,12], magnetic, and easy to functionalize with drugs, antibodies, or other molecular ligands, making them crucial for biomedical and clinical applications, including MRI [10]. The size, morphology, composition, and surface coating of MNPs determine their efficacy as contrast agents [13]. MNPs with diameters smaller than 50 nm have been shown to escape phagocytosis by the mononuclear phagocyte system and hence have longer circulation time than MNPs with diameters bigger than 50 nm. MNPs are coated with PEG to prevent agglomeration, reduce cytotoxicity, and prolong their circulation time. Faceted MNPs have been shown to demonstrate superior magnetism and relaxivities than spherical MNPs of similar size [13]. The superior magnetic properties of faceted MNPs make them better contrast agents for MRI than spherical MNPs. The size of the MNP core affects the anisotropy energy, relaxivity, and, consequently, the contrast in MRI images. T_2_-weighted gradient echo images of MNPs create negative contrasts, which increase with increasing particle size [13].

Targeted MNPs have been investigated as potential contrast agents for MRI for the early diagnosis of tumors [9,11,12,14,15]. Tumor xenografts targeted with MNPs and conjugated MNP systems showed greater tumor contrast enhancement than untreated systems. The LHRH receptor is expressed on the surface of more than 50% of TNBC cases, and these receptors have been targeted for treatment. Hu et al. [16] investigated the use of MNPs and LHRH-conjugated spherical-shaped MNPs for use as a contrast agent for the MRI of TNBC. The MNPs and LHRH-conjugated MNPs exhibited concentration-dependent hyper- and hypointensities on T_1_- and T_2_-weighted maps, respectively.

This paper presents the results of a study examining how MNP shape affects outcomes and the influence of LHRH-conjugated MNPs on T_2_-weighted MRI contrast enhancement in TNBC xenografts. Our research group previously examined in vitro T_1_- and T_2_-weighted MRI scans using different concentrations of MNPs and LHRH-MNPs as contrast agents [16]. The study confirmed that MNPs and LHRH-MNPs can potentially function as T_2_-based contrast agents. Prior work by our research group has led to the successful conjugation of the LHRH peptide to the surfaces of PEG-coated spherical magnetite nanoparticles (MNPs) for use as contrast agents in in vivo MRI of TNBC xenografts [5,16]. The MNPs were PEGylated to achieve improved monodispersity, stability, biocompatibility, and longer retention times [5,17,18]. LHRH-PEG-coated spherical MNPs successfully targeted TNBC cells [5,17,19]. Meng et al. [17] reported that the use of LHRH-superparamagnetic iron oxide nanoparticles enhanced the MRI contrast of TNBC in T_2_-weighted images. We hypothesize that, given the same condition and concentration of MNPs, nanorod-shaped MNPs will yield better-resolved MRI scans that can clearly detect TNBC tissue than spherical-shaped MNPs because elongated nanostructures (nanowires and nanorods) are shown to be efficient MRI contrast agents, having prolonged retention time at the tumor site, and increased specific attachment to their target during drug delivery compared to spherical nanoparticles [10]. The enhanced MRI contrast properties of nanorods are attributed to the higher surface area anisotropic morphology, which induces a more substantial magnetic field perturbation over a larger volume, more effectively for the sphere protons [20]. Guo et al. [21] have shown that biomimetic nanoparticle systems, such as erythrocyte membrane-coated gold nanorods, demonstrate enhanced biocompatibility, tumor targeting, and imaging-guided therapeutic efficacy. Their results underscore the promise of multifunctional nanoplatforms for integrated cancer diagnosis and treatment.

In this study, two different shapes of MNPs, spherical-shaped MNPs and nanorod-shaped MNPs, were synthesized, PEGylated, and functionalized with LHRH to target the overexpressed LHRH receptors on the surface of the TNBC. The MNPs serve as MRI contrast agents for TNBC xenografts. The MNPs are administered intravenously into nude mice via the tail vein. PEGylation of the MNPs enhances their transport in nude mice and prevents their clearance from the bloodstream before they reach TNBC xenografts [16,18]. The MRI scans of tissues injected with spherical and nanorod MNPs are presented, along with the implications for early TNBC detection.

## 2. Methods

### 2.1. Synthesis of Spherical MNPs

The co-precipitation method of magnetite nanoparticle synthesis was used to synthesize spherical MNPs [22,23]. In this method, 0.1 M iron (II) sulphate heptahydrate (Sigma Aldrich, St. Louis, MO, USA) and 0.2 M iron (III) chloride hexahydrate (Sigma Aldrich, St. Louis, MO, USA) were dissolved in 100 mL of 0.1 M hydrochloric acid (Thermo Fisher Scientific, Waltham, MA, USA) in a round-bottom flask under a constant nitrogen gas supply that was used to maintain an inert atmosphere. The flask was heated on a magnetic stirrer set at 80 °C throughout the process. This reaction was followed by the dropwise addition of 70 mL of 2 M sodium hydroxide under vigorous stirring at room temperature for 40 min. The black precipitates were washed by suspending them in 40 mL of deionized water, vortexing at 100 rpm, and then centrifuging at 4500 rpm for 15 min, 4 times. The supernatant after each wash was discarded, and fresh 40 mL of deionized water was used at the beginning of each wash. The final supernatant, which was clear and debris-free by the fourth wash, was discarded, and the MNPs were air-dried and stored at 4 °C for future use.

### 2.2. Synthesis of Rod-Shaped MNPs

Nanorod-shaped MNPs were synthesized in two steps following the method described by Adhikari et al. [10]. The first step yielded iron oxyhydroxide (FeOOH) nano-needles, and the second yielded nanorod-shaped MNPs from the magnetite nanoneedle. To synthesize the nano-needles, 20 mmol of iron (III) chloride (Sigma-Aldrich, St. Louis, MO, USA) was dissolved in 100 mL of deionized water (DI) at 25 °C in a loosely fitted single-neck flask.

A 33.3% (*v*/*v*) stock solution of polyethyleneimine (PEI) (Sigma-Aldrich, St. Louis, MO, USA) was prepared by dissolving 20 mL of PEI in DI to a final volume of 60 mL. Subsequently, 1.5 mL of the prepared stock solution was added to the single-neck flask containing deionized water and iron (III) chloride. The resulting solution was stirred at 400 rpm using a magnetic stirrer (Benchmark Scientific Inc., Sayreville, NJ, USA) at 80 °C. The experiment was performed in an oil bath heated to 80 °C on a magnetic stirrer for 2 h. It yielded iron oxyhydroxide nano-needles suspended in solution as brownish-yellow precipitates.

The precipitates were washed five times with ethanol and centrifuged at 4500 rpm for 15 min after each washing. The resulting samples were then dried in a vacuum desiccator for 48 h. The dried nanoneedles were placed in a centrifuge tube and stored at 4 °C. The second step involves converting iron (III) oxyhydroxide into nanorod-shaped MNPs. First, a three-neck round-bottom flask was filled with 20 mL of DI and 0.1 g of iron (III) oxyhydroxide. An inert atmosphere was established in the flask by flowing argon for 10 min. The mixture was then stirred at 400 rpm with a magnetic stirrer while dropwise adding 250 µL of hydrazine as a reducing agent.

The resulting mixture was heated at 90 °C for 15 h (under continuous stirring) to form a black precipitate, indicative of iron (II, III) oxide (magnetite). The latter was separated by centrifuging at 4500 rpm for 15 min and washed five times with deionized water. It was then dried in an airflow chamber and stored at 4 °C.

### 2.3. Characterization of Synthesized Magnetite Nanoparticles

#### 2.3.1. Ultraviolet-Visible Spectrophotometry

An Ultraviolet-Visible (UV-Vis) spectrophotometer (UV-1900, Shimadzu Corporation, Tokyo, Japan) was used to obtain the absorbance of the MNPs. Deionized water was used as the reference sample. The MNPs were dispersed in deionized water and then serially diluted to a concentration with measurable absorbance in the UV-Vis spectrophotometer. The peak absorbances obtained for the different types of MNPs synthesized were compared with those reported in the literature [16,24,25].

#### 2.3.2. Fourier Transform Infra-Red Spectroscopy

The MNPs were dispersed in deionized water, and two drops of the dispersed nanoparticles were mounted on a sample holder of the Fourier Transform Infra-Red (FTIR; IRS Spirit, Shimadzu, Columbia, MD, USA). The FTIR transmittance values were obtained following the manufacturer’s protocol. Transmittance values obtained from FTIR were used to confirm the presence of MNPs by comparing the measured transmittance values with the standard transmittance values of MNPs, PEGylated MNPs, and LHRH-conjugated MNPs published in the literature [24,25].

#### 2.3.3. Transmission Electron Microscopy (TEM)

The synthesized MNPs were dispersed in deionized water. A very tiny drop of the dispersed solution was placed on Cu grids and allowed to dry. The samples were then viewed with Transmission Electron Microscopy (TEM: Philips CM10, Amsterdam, The Netherlands) at operating voltages (60 and 100 kV), and high-resolution images of the MNPs were taken for further analysis.

#### 2.3.4. Polydispersity and Zeta Potential Measurement

The polydispersity index and zeta potential of the MNPs were measured to assess their heterogeneity and surface charge. Samples were measured by dispersing 3 mg of the nanoparticles in 1 mL of deionized water under sonication for 30 min. Subsequently, 200 µL of the suspension was loaded into a folded capillary zeta cell (Malvern Instruments Ltd., Malvern, UK) and analyzed using a Zetasizer Nano ZS (Malvern Instruments Ltd., Malvern, UK).

### 2.4. Conjugation of Luteinizing Hormone-Releasing Hormone to MNPs

The surfaces of the synthesized MNPs were first coated with a combination of CT(PEG)_12_ (carboxy-PEG_12_-thiol) and MT(PEG)_4_ (methyl-PEG_4_-thiol) (Thermo Scientific, USA) according to the manufacturer’s instructions to prevent clearance of the MNPs from the bloodstream via phagocytosis. The surfaces of the PEGylated MNPs were functionalized with LHRH to facilitate binding to and attachment to LHRH receptors, which are more abundant on triple-negative breast cancer (TNBC) cells and tissues. PEGylation and conjugation were performed according to the protocol developed by Obayemi et al. [16]. The amide group in the PEG forms a covalent bond with one of the two carbonyl groups on glutaraldehyde, and the amino group in LHRH forms another covalent bond with the second carbonyl group on glutaraldehyde.

Briefly, 0.5 mL of 1 mg/mL PEG-coated MNPs were dispersed in 0.25 mL of deionized water in a conical flask by sonication under nitrogen gas. Subsequently, 50 µL of glutaraldehyde (Sigma-Aldrich, USA) was added to the PEG-coated MNPs solution, and the mixture was incubated overnight at room temperature (25 °C). The resulting solution was purified three times using a 100 kDa spin column and deionized water. The purified glutaraldehyde-activated PEG-coated MNPs were resuspended in 0.3 mL of deionized water. Subsequently, 0.1 mL of a 0.1 mg/mL LHRH solution (Bachem Americas Inc., Torrence, CA, USA) was added to the resuspended particles and left overnight at 4 °C under continuous stirring on a magnetic stirrer. The solution was passed through a 50 kDa molecular weight spin column to eliminate unbound LHRH ligands. The MNPS were resuspended in 0.2 mL of deionized water and stored at 4 °C in a new vial.

Four types of MNPs were made for this study: non-functionalized nanorod-shaped MNPs (BMNR); LHRH-conjugated nanorod-shaped MNPs (LCMNR); non-functionalized spherical-shaped MNPs (BSSMNP); and LHRH-conjugated spherical-shaped MNPs (LCSSMNP).

### 2.5. Characterization of PEGylated and Conjugated MNPs and LHRH-MNPs

The PEGylated MNPs and LHRH-conjugated MNPs were characterized using UV-Vis and Fourier Transform Infrared Spectroscopy.

#### 2.5.1. Ultraviolet Visible Spectrophotometry

An ultraviolet-visible spectrophotometer (UV-VIS 1900, Shimadzu, USA) was used to obtain the absorbance of PEGylated MNPs (BMNR and BSSMNP) and the LHRH-conjugated MNPs (LCMNR and LCSSMNP). Deionized water was used as the reference sample. The peak absorbances obtained were compared with those reported in the literature [16] for PEGylated and LHRH-conjugated MNP.

#### 2.5.2. Fourier Transform Infrared Spectroscopy

PEGylation of the MNPs and their conjugation to LHRH were validated by Fourier Transform Infrared Spectroscopy (FTIR) analysis of the samples. The validation was done by comparing the FTIR values obtained to standard transmittance values for PEGylation of MNP and conjugation of LHRH to MNPs published in the literature [16].

### 2.6. Triple-Negative Breast Cancer Tumor Model

Triple-negative breast cancer (TNBC) MDA-MB-231 cells were procured from American Type Culture Collection (ATCC), Manassas, VA, USA. These MDA-MB-231 cells were grown in L-15 base media supplemented with 10 mcg/mL penicillin, 100 mcg/mL streptomycin, and 10% FBS at 37 °C under atmospheric pressure. The TNBC cells were grown in a Matrigel suspension.

The Jackson Laboratory (Bar Harbor, ME, USA) provided 15 nude mice (7 weeks old) for this study. The animals used in the current work were approved for use by the University of Massachusetts Medical School Institutional Animal Care and Use Committee (UMMS IACUC). All animal experiments were performed in accordance with a protocol approved by the UMMS IACUC (IACUC ID: AMEND202100328) in Worcester, MA. Each mouse was injected subcutaneously with 1 × 10^7^ MDA-MB-231 cells in the back to induce a TNBC tumor. The procedure adopted here follows the approved IACUC protocol. Tumor cells were injected into 14 of the 15 mice. After tumor injection, the mice were monitored daily for three weeks. The tumor’s longest dimension, its shortest perpendicular dimension, and weight were measured weekly using calipers. The average tumor volume at week 3 was 105.7 mm^3^.

### 2.7. Magnetic Resonance Imaging of Tumor-Bearing and Non-Tumor-Bearing Mice

The MRI procedure was performed under inhalation anesthesia. A 4–5% concentration of isoflurane was used for induction of anesthesia, and 2% was used for maintenance. The mice were secured in the animal bed and placed on a coil compatible with a physiological monitoring system. MRI scans of mouse trunks with TNBC tumors and without TNBC tumors were obtained. MRI was performed on a 3 Tesla system ( Ingenia CX dStream 3.0T, Philips Healthcare, Best, The Netherlands) and a 7 Tesla system (Bruker BioSpec 70/30 USR horizontal-bore MR system, Bruker BioSpin, Ettlingen, Germany). Pre-contrast whole-body MRIs were obtained using T_2_-weighted scans as a baseline. Afterwards, an aliquot (200 μL) of 240 mg MNPs kg^−1^ (mouse weight) was injected intravenously into the mice through the tail veins. The average mouse weight used for the study was 22.7 ± 1.7 g. The typical dose of MNPs for MRI is ~18–70 µmol Fe/kg body weight [12,26]. Previous studies have shown that 250 mg/kg of mouse body weight is safe and sufficient for in vivo MRI [11]. The nude mice used in this research were divided into five groups. The first four groups consisted of three mice each, with induced TNBC tumors. The first group was injected with BMNR, the second with LCMNR, the third with BSSMNP, and the fourth with LCSSMNP. The fifth group consisted of two mice induced with TNBC tumors and one without TNBC tumors. The fifth group served as controls and was not injected with any MNPs. The aliquots of MNPs were injected into the mice without removing the mice from the MRI holder.

The mice injected with MNPs and the controls were scanned at 3 Tesla (3T) and 7 Tesla (7T) MRI pre- and post-2 h after nanoparticle injection, using the T_2_-weighted MRI protocol employed in prior work by our research group [5,16]. The MRI scanning parameters for the T_2_-weighted imaging were as follows: Echo Time (TE) = 80 ms; Repetition Time (TR) = 5500 ms; Field of View (ap,fh,rl) = 30 × 30 × 22; Scan resolution (x, y) = 300 × 288. Number of Slices = 22; Thickness = 1.0 mm. A previous study showed that T_2_-weighted scans of the MDA-MB-231 xenograft tumor, obtained 2 and 24 h post-intravenous injection of MNPs and LHRH-MNPs, demonstrated significant negative enhancement for both MNPs and LHRH-MNPs at 2 h post-injection [5]. The T_2_-weighted scans of tumor xenografts from mice injected with LHRH-MNPs showed significant negative enhancement at 24 h post-injection. MRI was performed on the same animal at the same position, using identical scanning sequences. Body temperature and respiratory rate were monitored throughout the scan. After scanning, the animals were observed and allowed to recover at room temperature. The mice were sacrificed after recovery. The tumors, kidneys, lungs, liver, and spleen were removed from the sacrificed mice and snap-frozen at −80 °C to prepare for histological analysis.

### 2.8. MRI Image Analysis

The MR images obtained with 3T and 7T MRI were saved in DICOM format and viewed using DICOM Viewer software (RadiAnt DICOM viewer (64-bit). Ink Version 2021, Medixant, Poznan, Poland, Eastern Europe) and Fuji (Image J; National Institute of Health (NIH), Bethesda, MD, USA). The MR images were evaluated based on signal intensity. The MRI slices that best revealed the breast tumor’s resolution before and after MNP injection were selected as the region of interest (ROI). The ROI radius was set to the size of the largest tumor, excluding the background. The measurement tool features in Fuji and DICOM Viewers were used to take measurements (area, mean signal intensity, standard deviation, minimum, and maximum signal intensity, pixel values) under the defined ROI. The difference in MRI signal intensity of the ROI was calculated. A background-subtraction tool feature of Imaje J was used to correct for uneven background.

The signal-to-noise ratio, which quantifies the signal of the ROI (TNBC xenograft tissue) relative to the background noise, was calculated using the following expression:

(1) SNR = (SI_ROI_ − SI_background_)/SD_background noise_,

where SI_ROI_ is the mean signal intensity (SI) of the ROI, and SD is the standard deviation of the background noise.

The contrast-to-noise ratio (CNR), which assesses the efficiency of contrast enhancement of the TNBC xenograft tissue in relation to the background noise, was calculated using the following expression:

(2) CNR = |SIA − SIB|/SD_background noise_,

where tissue SIA is the signal intensity of the ROI post injection with MNPs, and SIB is the signal intensity of the ROI pre-injection with MNPs.

### 2.9. Immunohistochemistry Staining

The organs preserved from mice sacrificed after the scans were embedded in Optimal Cutting Temperature (OCT) compound (Fisher Scientific, USA). They were then held in a fixed position with OCT and cut into 20 µm compound sections using a cryostat (Leica, Wetzlar, Germany). Histology was performed on excised tumors using immunohistochemical staining. Prussian blue staining was used to confirm nanoparticle uptake, while hematoxylin and eosin (H&E) staining was used to identify tumor necrosis, histological changes in vital organs, and cytotoxicity. This immunological staining was performed according to the protocols established in our prior research. TEM images of the tumors harvested from the mice sacrificed after MNPs injection were also taken for further analysis, following established protocols described in earlier work from our research group [5]. The Prussian Blue Cell Staining Reagent Pack (Sigma-Aldrich) was used to stain the thin tissue sections according to the manufacturer’s instructions. The stained tissues were viewed using a Nikon Eclipse TE2000 (Nikon, Tokyo, Japan) bright-field microscope.

### 2.10. Ethics Statement

The studies adhered to the NIH guidelines for the care and use of laboratory animals (NIH Publication No. 85-23, Rev. 1985).

### 2.11. Statistical Analysis

Origin Lab 2026, 64-bit Ink was used to plot the FTIR and UV results. The Origin Lab statistical software package (2026) was also used to compare the mean contrasts and standard deviation of T_2_-weighted MRI scans (obtained using the RadiAnt DICOM viewer Ink Version 2021), using one-way ANOVA with post hoc Fisher’s Least Significant Difference (LCD) test at the 95% confidence level. The values obtained were considered statistically significant at *p* < 0.05.

## 3. Results

### 3.1. Nanoparticle Shapes and Size Distribution

**Figure 1:**
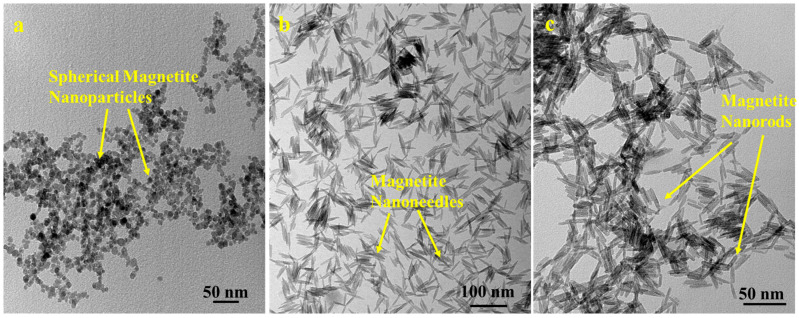
TEM photomicrographs of (**a**) spherical-shaped MNPs, (**b**) magnetite nano-needles, and (**c**) nanorod-shaped MNPs (magnetite nanorods).

The synthesized spherical MNPs are 20 nm in diameter, while the magnetite nano-needles and magnetite nano-rods are 50 nm in length, each with an aspect ratio of 10. Figure 1b shows nanoneedles formed in the first step of nanorod synthesis. The needle tip formed due to the unidirectional growth of MNPs. Also, Figure 1c shows the nanorods that formed when the nano-needles from the first step were reduced by hydrazine. The formation of magnetite nanorods resulted in the loss of needle tips. The polydispersity index (PDI) for BMNR, LCMNR, BSSMNP, and LCSSMNP was 0.06 ± 0.01, 0.08 ± 0.01, 0.07 ± 0.01, and 0.09 ± 0.01, respectively, indicating that the MNPs were narrow-dispersed and moderate-dispersed and did not aggregate. The zeta potential of BMNR, LCMNR, BSSMNP, and LCSSMNP was found to be 39.6 ± 0.4 mV, 55.9 ± 0.5 mV, 42.9 ± 0.3 mV, and 54.9 ± 0.6 mV, respectively. There is also a need to explore the conjugation efficiency and the hydrodynamic size of the MNP in future studies.

### 3.2. LHRH-Magnetite Nanoparticles PEGylation and Conjugation

**Figure 2:**
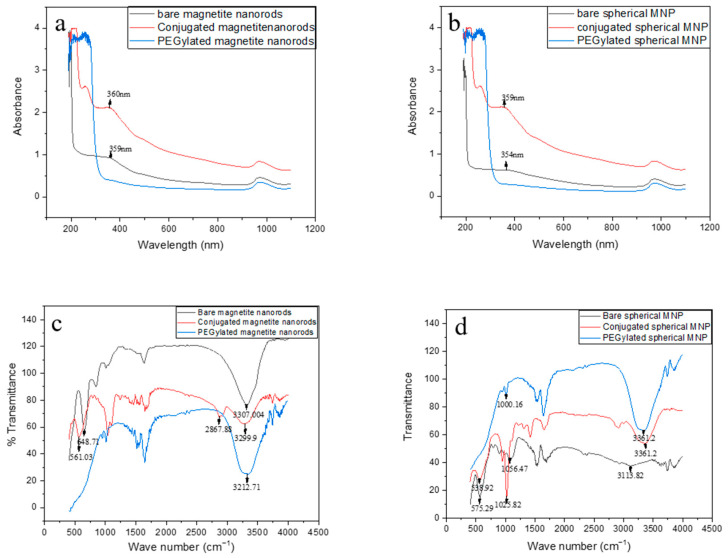
Physicochemical characterization of the synthesized magnetic nanoparticles (MNPs). (**a**) UV-Visible spectrophotometer of nanorod-shaped MNPs. (**b**) UV-Visible spectrophotometer of spherical-shaped MNPs. (**c**) FTIR transmittance spectra of nanorod-shaped MNPs. (**d**) FTIR transmittance spectra of spherical-shaped MNPs.

In Figure 2a, the UV-vis spectrophotometer shows that bare magnetite nanorods (not treated with PEG and not linked to LHRH) had peaks at 359 nm, while bare spherical-shaped MNPs in Figure 2b (also not treated with PEG and not linked to LHRH) had peaks at 354 nm. These values match the UV-visible spectrophotometric peak for the Fe-O bond reported in the literature [24]. After being coated with CT-PEG and MT-PEG (PEGylation), the Fe-O bond peak is hidden and cannot be seen in the UV. The Fe-O bond reappears after conjugation to LHRH at 360 nm for nanorods (Figure 2a) and 359 nm for spherical MNPs (Figure 2b). The conjugation-activated functional group shown in the plot is the Fe-O bond.

The FTIR spectra in Figure 2c ranged from 4100 cm^−1^ to 500 cm^−1^ for bare spherical-shaped MNPs, PEGylated spherical-shaped MNPs, and LHRH-conjugated spherical-shaped MNPs (Figure 2d). This range is consistent with the FTIR spectra of magnetite [27,28]. The transmission band at 575.29 cm^−1^ for bare-shaped MNPs and 538.92 cm^−1^ for LHRH-conjugated spherical-shaped MNPs is a characteristic of the Fe-O bond (Figure 2d).

There is no dip showing Fe-O stretching in PEGylated spherical-shaped MNP spectra because PEG effectively coats the MNPs. The dip at 1000.16 cm^−1^ for PEGylated spherical-shaped MNP and 1025.82 cm^−1^ for LHRH-conjugated spherical-shaped MNPs signifies carbon-oxygen bond stretching [27,28]. This measurement indicates the presence of CT-PEG and MT-PEG. The dips at 3113.82 cm^−1^, 3361.2 cm^−1^, and 3361.2 cm^−1^ indicate bond formation in water. LHRH-conjugated spherical-shaped MNPs showed additional signatures, which include 1670.97 cm^−1^ for NH_2_ stretching from LHRH and 2994.05 cm^−1^ for methylene stretching for MT PEG after activation.

The dips at 643.71 cm^−1^ and 3307.004 cm^−1^ in Figure 2c for bare nanorod-shaped MNPs indicate Fe-O bonding and bond stretching for water formation, respectively. The dip at 561.08 and 3299.9 for LHRH-conjugated nanorod-shaped MNPs (Figure 2c) is characteristic of the Fe-O bond and the bond for the formation of water, respectively [16].

### 3.3. TEM Characterization of Magnetite Nanoparticles in Tissues

**Figure 3:**
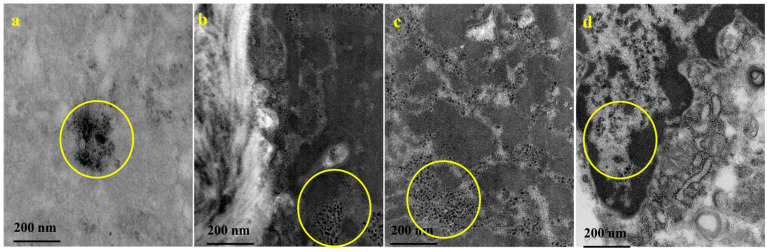
TEM micrographs showing the presence of MNPs in the TNBC tumor excised from the mice sacrificed after the MRI scanning. The types of MNPs encircled in yellow circles in the images are as follows: (**a**) BMNR, (**b**) LCMNR, (**c**) BSSMNP, and (**d**) LCSSMNP.

Figure 3 shows the ultrastructure of tumor tissues made of MDA-MB-231 breast cancer cells in nude mice, together with the MNPs injected via the bloodstream to reach the TNBC tumor tissues. The circled regions indicate the presence of magnetite nanoparticles in tissue, 2 h after intravenous magnetite nanoparticle injection.

### 3.4. T_2_-Weighted Magnetic Resonance Imaging

**Figure 4:**
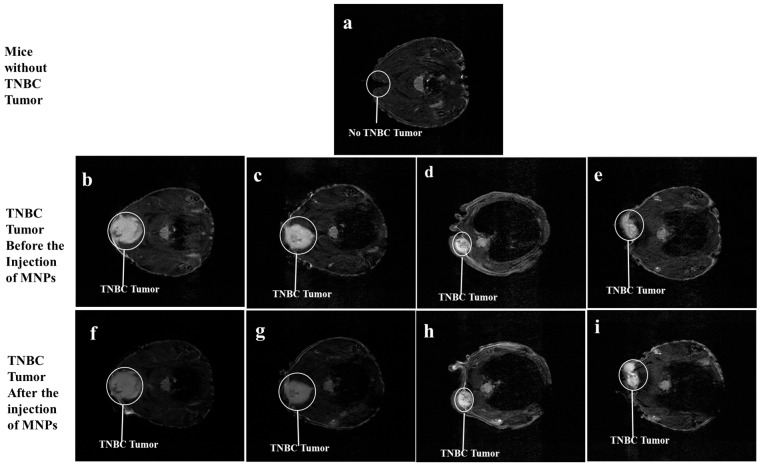
T_2_-weighted 3 Tesla MRI scans. (**a**) is the 3T MRI scans of mice before TNBC tumor induction. (**b**–**e**) are the 3 Tesla MRI scans of the TNBC tumor induced in nude mice. (**f**) is a 3 Tesla MRI scan of the TNBC tumor shown in (**b**), 2 h after the mice were injected with BMNR. (**g**) is a 3 Tesla MRI scan of the TNBC tumor shown in (**c**), 2 h after the mice were injected with LCMNR. (**h**) is a 3 Tesla MRI scan of the TNBC tumor shown in (**d**), 2 h after the mice were injected with BSSMNP. (**i**) is a 3 Tesla MRI scan of the TNBC tumor shown in (**e**), 2 h after the mice were injected with LCSSMNP.

Figure 4a is the dorsal view of mice without any induced tumors. Figure 4b–i show representative images of the sectional dorsal views of mice induced with TNBC tumors on their trunks, with the TNBC tumor appearing as a gray-colored irregular mass encircled in white. Figure 4b–e show the T_2_-weighted MRI scans of the TNBC tumor before the injection of MNPs, while the images in Figure 4f–i reveal the T_2_-weighted 3T MRI scans of the TNBC tumor tissue 2 h after the injection of the MNPs in the nude mice via the tail vein. The T_2_-weighted 3T MRI scans in Figure 4f and Figure 4g appear darker, 2 h post-injection of BMNR and LCMNR, respectively. The T_2_-weighted MRI scans in Figure 4h and Figure 4i show images taken 2 h after injection of BSSMNP and LCSSMNP, respectively.

**Figure 5:**
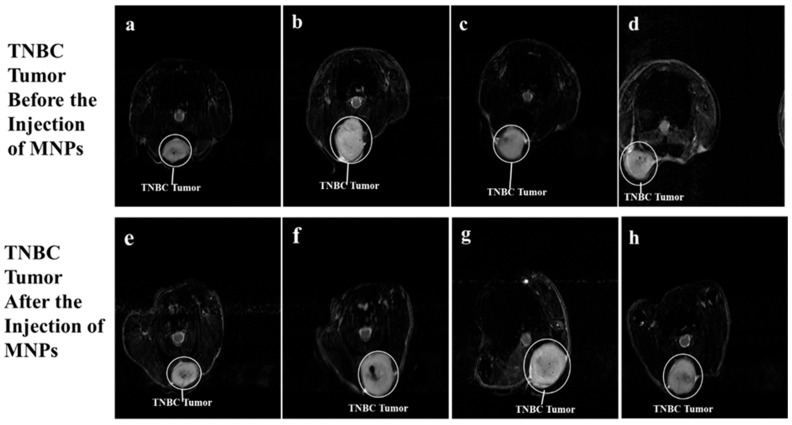
T_2_-weighted 7 Tesla MRI scans. (**a**–**h**) 7 Tesla MRI scans of TNBC tumors induced in nude mice. (**e**) is the 7 Tesla MRI scan of the TNBC tumor shown in (**a**) 2 h after the mice were injected with BMNR. (**f**) is the 7 Tesla MRI scan of the TNBC tumor shown in (**b**) 2 h after the mice were injected with LCMNR. (**g**) is the 7 Tesla MRI scan of the TNBC tumor shown in (**c**) 2 h after the mice were injected with BSSMNP. (**h**) is the 7 Tesla MRI scan of the TNBC tumor shown in (**d**) 2 h after the mice were injected with LCSSMNP.

Figure 5a–h show sectional dorsal views of mice induced with TNBC tumors on the trunk. The TNBC tumor appears as a white colored, irregularly shaped tumor. The T_2_-weighted 7T MRI scans of the TNBC tumors in Figure 5e, Figure 5f, Figure 5g, and Figure 5h appear darker, with traces of dark colorations on the TNBC tumor, 2 h post-injection of BMNR, LCMNR, BSSMNP, and LCSSMNP, respectively.

### 3.5. Immunohistochemical Staining for Magnetite Nanoparticles Uptake (Prussian) and Cytotoxicity (H&E)

#### 3.5.1. Prussian Blue Staining

**Figure 6:**
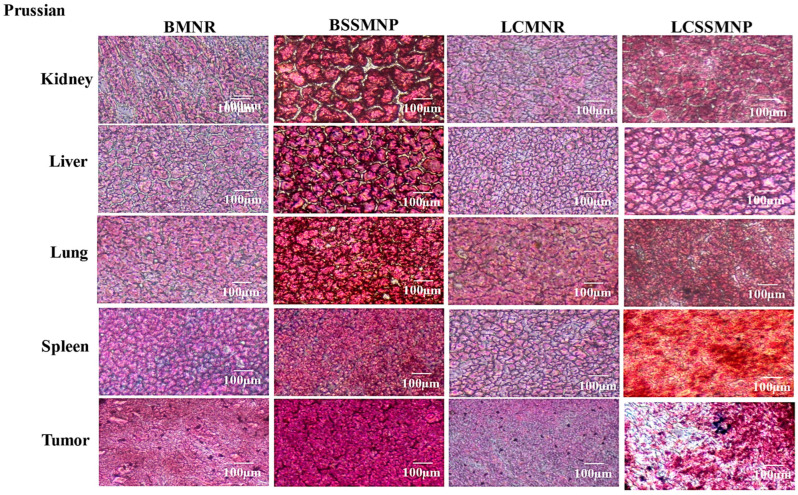
Prussian blue staining of various tissues of mice injected with different types of magnetite nanoparticles.

The Prussian blue stains MNPs purple when present in tissues. This study examined the presence of magnetite nanoparticles in the kidneys, liver, lungs, spleen, and tumor tissues in mice administered with MNPs. The organs in the mice were stained with Prussian Blue. The results show that no MNPs were detected in the liver, kidney, lung, or spleen after injection of BMNR, LCMNR, BSSMNP, or LCSSMNP into the mice (Figure 6). Prussian blue staining was observed in tumors after injection of BMNR, LCMNR, and LCSSMNP into mice (Figure 6).

#### 3.5.2. Hematoxylin and Eosin Staining

**Figure 7:**
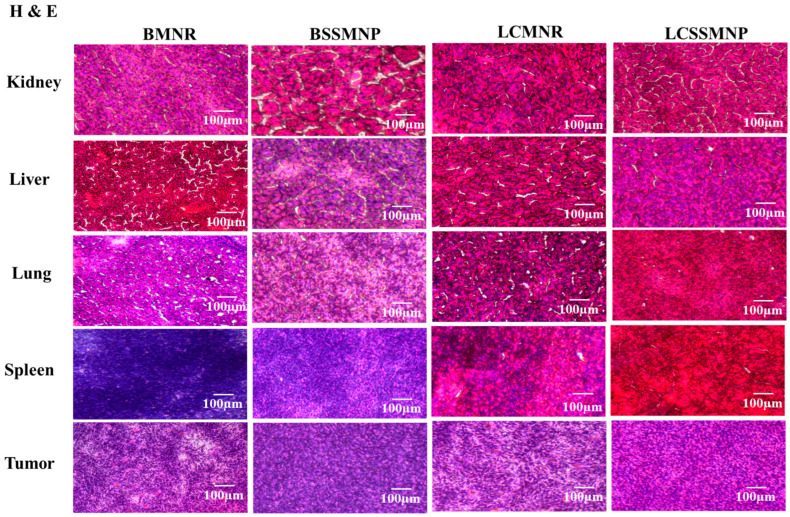
Hematoxylin and Eosin staining of tissues for mice injected with magnetite nanoparticles.

Hematoxylin (H) stains the cell nuclei purplish blue (or dark purple), while eosin (E) stains the extracellular matrix, for example, collagen and cytoplasm, pink. The H&E staining revealed that there was no evidence of cytotoxicity in the mice that were injected with magnetite nanoparticles (Figure 7). H&E staining of the kidneys, lungs, liver, and spleen revealed no sign of toxicity. However, the tumor tissues showed signs of necrosis when the magnetite nanorods were used. This finding is similar to what has been reported [29].

### 3.6. Effect of Magnetic Field Strength on Magnetite Resonance Imaging

**Figure 8:**
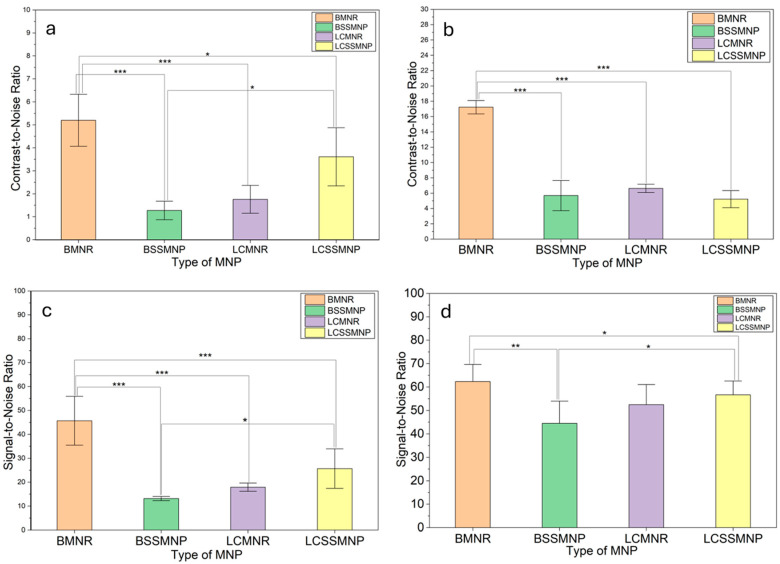
Contrast-to-Noise ratio (CNR) and Signal-to-Noise ratio (SNR) from the magnetite nanoparticles (MNPs) used as contrast agents at 3 Tesla (**a**,**c**) and 7 Tesla magnetic field strength (**b**,**d**). * means 0.01 < *p* < 0.05; ** means 0.001 ≤ *p* < 0.01; *** mean *p* < 0.001.

Figure 8 illustrates the contrast-to-noise ratio (CNR) and the signal-to-noise ratio (SNR) for the average contrast values obtained from 3T and 7T T_2_-weighted MRI scans, with a significance level of *p* < 0.05. The CNR of BMNR is significantly higher in comparison to those of LCMNR, BSSMNP, and LCSSMNP for the T_2_-weighted MRI scans conducted at both 3T (Figure 8a) and 7T (Figure 8b) field strengths. The CNR of LCSSMNP is significantly greater than that of BSSMNP for MRI scans acquired at 3T. The SNR of BMNR is significantly higher compared to LCMNR, BSSMNP, and LCSSMNP for the T_2_-weighted MRI scans performed at 3T (Figure 8c). Additionally, the CNR of LCSSMNP exceeds that of BSSMNP significantly at 3T field strength. At 7T field strength (Figure 8d), the SNR of BMNR is significantly higher than BSSMNP and LCSSMNP. The SNR of LCSSMNP is significantly higher than that of BSSMNP.

## 4. Discussion

This study compared the shape effects of MNP as a contrast agent on 3T and 7T T_2_-weighted MRI scans. The impact of LHRH conjugation on MNPs on T_2_-weighted MRI scans was also explored. The contrast agents shorten the T_2_ relaxation time and manifest as hypointense signals (having a darkening effect/decreased signal intensity) on T_2_-weighted MRI scans [30,31].

The results of this study revealed that contrast enhancement of TNBC tumor xenograft by BMNR was significantly greater than that of BSSMNP, LCMNR, and LCSSMNP at 3T (Figure 8a) and 7T (Figure 8b) field strength. The contrast enhancement of LCSSMNP for the TNBC tumor was also higher than that from BSSMNP at a 3T MRI scan (Figure 8a). This is similar to the previous report that LHRH-MNPs (LCSSMNP) produce greater signal changes and contrast for TNBC tumor imaging and detection than MNPs (BSSMNP) in T_2_-based MRI [5,25]. BMNR produced significantly better MRI signals and contrast than BSSMNP, LCMNR, and LCSSMNP at 3T (Figure 8a,c). The SNR value from the TNBC tumor xenograft when BMNR was used as the contrast agent was significantly higher than that from BSSMNP and LCSSMNP (Figure 8d) at 7T. Additionally, the SNR value with LCSSMNP as the contrast agent was significantly higher than that with BSSMNP (Figure 8d) at 7T. The higher the SNR and CNR values, the clearer the MRI image and the easier it is to detect the ROI (TNBC tumor xenograft). The CNR and SNR values were greater at 7T than at 3T field strength. This finding is consistent with previous reports that T_2_-weighted MRI scans taken at higher magnetic field strengths produce better-resolved images than scans taken at lower field strengths [15].

The LHRH on the LCSSMNP, the specific targeting moiety for the TNBC tumor xenograft, may have influenced LCSSMNP uptake into the tumor compared with the BSSMNP. Previous studies have shown that LHRH enhances SPION/MNP uptake in TNBC tumor xenografts, thereby improving spatial resolution in T_2_^-^weighted MRI scans when LHRH-SPIONs and LHRH-MNPs are used as MRI contrast agents [5,17]. The lower CNR and SNR values for LCMNR compared to LCSSMNP may be due to the concentration that have penetration into the TNBC tumor xenograft. The presence of the BMNR, LCMNR, BSSMNP, and LCSSMNP in the tumor tissue within 2 h of MNP injection is shown on the TEM micrograph (Figure 3a,b). The relaxation effects of MNPs contrast agents are influenced by their local concentration, applied field strength, and the environment in which the contrast agents interact with the surrounding protons [32].

Prussian blue staining is reported to be reliable for detecting iron oxide in tissue sections [33,34]. Prussian blue stains revealed that MNPs were present in the TNBC tumor in mice injected with BMNR, LCMNR, and LCSSMNP (Figure 6). These nanoparticles observed in tumor tissues may have penetrated the tumor due to enhanced membrane permeability, the leaky vasculature characteristics of cancer cells, and the presence of LHRH [35]. This targeting moiety binds to the overexpressed LHRH receptors on the surface of TNBC cells. Previous research suggests that LHRH-SPIONS may have accumulated in the TNBC cells via receptor-mediated endocytosis [11]. Significant evidence of iron oxide staining was observed in the tumor tissues extracted from mice injected with BMNR and LCMNR (Figure 7). However, a reduced incidence of MNP (iron oxide) staining was observed in tumor tissues after injection with LCSSMNP. No stain confirming the presence of MNPs was observed in tumor tissues injected with BSSMNP (Figure 7). H&E staining revealed no signs of toxicity in the kidneys, liver, lungs, or spleen. There were also signs of necrosis in the tumor tissues injected with BMNR and LCMNR (Figure 7).

Thus, the results obtained from this study showed that nanorod-shaped MNPs (BMNR) are better MRI contrast agents than spherical-shaped nanoparticles (BSSMNP and LCSSMNP). BMNR also produced a greater contrast and MRI signal than LCMNR. The smaller size of the BMNR compared to the LCMNR may be responsible for this. The signal intensity and contrast enhancement on MRI scans were greater at 7 Tesla than at 3 Tesla.

## 5. Conclusions

This study investigated the shape effects of MNPs used as contrast agents for the early detection of TNBC. BMNR produced more contrast in TNBC xenograft tissues than BSSMNP and LCSSMNP, at 3 Tesla and 7 Tesla, respectively. The CNR and SNR in TNBC xenograft tissues on T_2_-weighted MRI scans when BMNR was used as a contrast agent were much higher than when BSSMNP and LCSSMNP were used at a 3 Tesla field strength. MRI scans at 7 Tesla gave better-resolved T_2_-weighted MR images than those at 3 Tesla. Early diagnosis of TNBC using nanorod-shaped MNPs and higher magnetic field strengths (7 Tesla versus 3 Tesla) may enable earlier detection in patients than spherical MNPs and LHRH-conjugated MNPs currently used for MRI.

## Data Availability

The original contributions presented in the study are included in the article, further inquiries can be directed to the corresponding author.
